# Discapacidad y participación en actividades de ocio activo: resultados de una encuesta poblacional chilena

**DOI:** 10.1590/0102-311XES007724

**Published:** 2024-08-26

**Authors:** Nicole Chávez-Cunti, J. Jhonnel Alarco

**Affiliations:** 1 Disability Epidemiology Research Group, Universidad Científica del Sur, Lima, Perú.

**Keywords:** Actividades Recreativas, Personas con Discapacidad, Grupos de Población, Análisis de Datos Secundarios, Leisure Activities, Disabled Persons, Population Groups, Secondary Data Analysis, Atividades de Lazer, Pessoas com Deficiência, Grupos Populacionais, Análises de Dados Secundários

## Abstract

Las actividades recreativas son necesarias para mejorar la calidad de vida y el buen estado de salud de la población. Algunos estudios en países desarrollados han descrito que las personas con discapacidad participan menos en actividades recreativas. El objetivo de este estudio fue estimar la asociación entre la discapacidad y la participación en actividades de ocio activo en la población de 18 o más años de Chile, durante el año 2015. Se efectuó un estudio transversal analítico con los datos de la *II Encuesta Nacional de la Discapacidad* (ENDISC II) 2015 de Chile. La variable independiente fue la discapacidad y la variable dependiente fue la participación en actividades de ocio activo en los últimos seis meses. Se elaboraron modelos de regresión de Poisson y se estimaron razones de prevalencia (RP) con sus intervalos de 95% de confianza (IC95%). Se incluyeron a 12.236 participantes. Los chilenos con discapacidad moderada y severa tuvieron menos probabilidades de participar en actividades de ocio activo (RP = 0,96; IC95%: 0,93-0,99 y RP = 0,78; IC95%: 0,72-0,84, respectivamente), en comparación con los chilenos sin discapacidad. Cuando se estratificó por grupos de edad, esta asociación se mantuvo significativa solo en los mayores de 45 años. En conclusión, las personas con discapacidad de Chile participan menos en actividades de ocio activo en comparación con las personas sin discapacidad, aunque solo si son mayores de 45 años. Los programas sociales de recreación deberían priorizar la incorporación de personas mayores con discapacidad.

## Abstract (en)

Leisure activities are necessary to improve the quality of life and good health of the population. Some studies in developed countries have described that people with disabilities participate less in leisure activities. The aim of this study was to estimate the association between disability and participation in active leisure activities in the population aged 18 years or older in Chile, during 2015. An analytical cross-sectional study was conducted using data from the Chilean *II National Survey on Disability* (ENDISC II) 2015. The independent variable was disability, and the dependent variable was participation in active leisure activities in the last six months. Poisson regression models were developed and prevalence ratios (PR) and the 95% confidence intervals (95%CI) were estimated. In total, 12,236 participants were included. Chileans with moderate and severe disability were less likely to participate in active leisure activities (PR = 0.96; 95%CI: 0.93-0.99 and PR = 0.78; 95%CI: 0.72-0.84, respectively), compared to Chileans without disability. When stratified by age group, this association remained significant only in those older than 45 years. In conclusion, people with disabilities in Chile participate less in active leisure activities compared to people without disabilities, although only if they are older than 45 years. Social recreation programs should prioritize the incorporation of older people with disabilities.

## Resumo

Atividades recreativas são necessárias para melhorar a qualidade de vida e a saúde da população. Alguns estudos em países desenvolvidos relataram que as pessoas com deficiência participam menos de atividades recreativas. O objetivo deste estudo foi estimar a associação entre deficiência e participação em atividades de lazer ativas na população com 18 anos ou mais no Chile, em 2015. Foi realizado um estudo transversal analítico usando dados da *II Pesquisa Nacional sobre Deficiência* (ENDISC II), 2015, no Chile. A variável independente foi a deficiência e a variável dependente foi a participação em atividades de lazer ativas nos últimos seis meses. Foram criados modelos de regressão de Poisson e estimadas as razões de prevalência (RP) com seus intervalos de 95% de confiança (IC95%). Um total de 12.236 participantes foi incluído. Os chilenos com deficiência moderada e grave tinham menor probabilidade de participar de atividades de lazer ativas (RP = 0,96; IC95%: 0,93-0,99 e RP = 0,78; IC95%: 0,72-0,84, respectivamente) em comparação com os chilenos sem deficiência. Quando estratificada por faixa etária, essa associação permaneceu significativa apenas para aqueles com mais de 45 anos de idade. As pessoas com deficiência no Chile participam menos de atividades de lazer ativas em comparação com as pessoas sem deficiência, mas somente se tiverem mais de 45 anos. Os programas de recreação social devem priorizar a inclusão de pessoas mais velhas com deficiência.

## Introducción

Las actividades recreativas están conformadas por acciones agradables como divertirse, entretenerse, gozar, obtener felicidad y placer; son practicadas durante el tiempo libre y por interés propio ^1^. En la actualidad, las actividades de recreación han adquirido valor social y educativo desde el punto de vista personal y colectivo, ya que mejoran la salud, propician valores morales y favorecen la calidad de vida de las personas ^2^.

La recreación constituye, un factor importante para la realización del sujeto en cada una de las diversas etapas de su existencia. Estas actividades recreativas (también llamadas de ocio) son tareas que se realizan en casa o en la comunidad, y según la Organización Panamericana de la Salud (OPS) mejoran las funciones cardiorrespiratorias, musculares, salud ósea y reducen el riesgo de enfermedades no transmisibles y de depresión ^3^. Al respecto, numerosos estudios ^4^ ^,^ ^5^ ^,^ ^6^ han demostrado sus beneficios físicos y mentales en las personas. No obstante, las actividades de ocio pasivo (ver televisión, jugar videojuegos, usar la computadora, hablar por teléfono, etc.) son actividades sedentarias con limitados beneficios para la salud, mientras que las actividades de ocio activo (practicar un deporte, hacer ejercicio, ser voluntario o los viajes) favorecen la satisfacción con la vida ^7^. La actividad física de tiempo libre es aquella que no es esencial para la vida diaria y se realiza a criterio de la persona, e incluye la práctica de deportes, ejercicio y caminatas recreativas ^8^.

Según el *Informe Mundial de la Discapacidad 2011* ^9^, más de mil millones de personas en el mundo tienen algún tipo de discapacidad y alrededor de 200 millones de ellas tienen dificultades funcionales. La prevalencia de la discapacidad es mayor en los países en desarrollo, y la quinta parte de la población mundial (190 millones) está afectada por diferentes discapacidades ^9^. En Latinoamérica y el Caribe, alrededor de 70 millones de personas tienen alguna discapacidad ^10^. En Chile, según el *II Estudio Nacional de la Discapacidad* (ENDISC II) realizado entre junio y septiembre del 2015, se estima que 2,83 millones de personas se encuentran en situación de discapacidad ^11^.

La poca evidencia disponible indica que las personas con discapacidad tienen menor participación en actividades de ocio; además, estos estudios se han realizado en países desarrollados y primordialmente en adultos mayores ^12^ ^,^ ^13^. Por lo tanto, es necesario tener información en Latinoamérica sobre esta posible asociación. Los resultados de este estudio servirán para incrementar la evidencia sobre el vínculo entre la discapacidad y la participación en actividades recreativas, necesarias para mejorar la calidad de vida y el buen estado de salud, en las personas con o sin discapacidad.

El objetivo primario del estudio fue estimar la asociación entre la discapacidad y la participación en actividades de ocio activo en la población de 18 o más años de Chile durante el año 2015. Se planteó la hipótesis de que las personas con discapacidad tienen menor participación en actividades recreativas que las personas sin discapacidad. Asimismo, como objetivos secundarios, se propuso estimar esta asociación según grupos de edad y evaluar las diferencias en los diferentes tipos de ocio activo según discapacidad.

## Métodos

### Diseño y población

Se realizó un análisis transversal de los datos secundarios de la ENDISC II realizado en Chile en el 2015, cuya población estuvo conformada por adultos de 18 a más años y por niños de 2 a 17 años ^11^.

### Contexto

La ENDISC II fue realizada por el Ministerio de Desarrollo Social de Chile y su objetivo fue “*determinar la prevalencia y caracterizar la discapacidad a nivel nacional, identificando las principales brechas de acceso a las personas en situación de discapacidad y a partir de ello, evaluar los resultados en la aplicación de la normatividad vigente al respecto a nivel nacional e internacional*” ^11^. El tamaño de muestra de la ENDISC II incorporó a 11.981 viviendas, lo que representa 12.265 hogares. Se incluyeron 12.265 adultos de más de 17 años y a 5.515 niños de 2 a 17 años. Asimismo, se utilizó la técnica del muestreo aleatorio con 237 estratos muestrales, para lograr una representatividad nacional, urbana y rural de las 15 regiones de Chile. El muestreo fue estratificado porque respondió a los criterios de identificación de las personas en situación de discapacidad por extensión de territorio (región, zona), edad, género y quintil ^14^.

### Criterios de selección

Para cumplir con el objetivo del estudio se incluyeron a personas adultas de 18 o más años que participaron en la ENDISC II y se excluyeron las personas con datos incompletos o incongruentes.

### Variables

La variable independiente fue la discapacidad, que fue calculada por la propia ENDISC II. Esta variable se encuentra disponible en la base de datos y tiene tres categorías: sin discapacidad, discapacidad leve y moderada, y discapacidad severa. La metodología usada por la ENDISC II para medir la discapacidad en Chile se realizó según el modelo de crédito parcial generalizado (*generalized partial credit model* -GPCM) que es “*un modelo politómico de respuesta al ítem que incluye un parámetro de discriminación para cada ítem, y que consiste en construir una métrica de capacidad que va desde 0 (sin dificultad) a 100 (máxima dificultad)*” ^14^. Además, fue utilizada la puntuación media de la información proporcionada por las personas respecto de las dificultades en los dominios de funcionamiento, los mismos que fueron considerados en la escala para la determinación de prevalencia de discapacidad.

La variable dependiente fue la participación en actividades de ocio activo en los últimos seis meses, que se midió con la pregunta “¿Realizó o asistió a las siguientes actividades o lugares?, las cuales fueron: ir al cine, teatro, espectáculos/recitales de música popular, clásica, lírica, ballet, a museos o exposiciones, eventos deportivos, festividades o eventos locales, parques/jardines, vacaciones, actividades al aire libre (acampar, equitación, visitas turísticas, otras actividades), festividades o actividades religiosas, reuniones sociales o familiares, restaurantes, bares, discotecas, pubs o ‘salió de fiesta’, ‘a vitrinear’ (tiendas, persas, malls, mercados u otros), de paseo (dentro o fuera de su localidad)”. Se asumió que la persona participó en actividades de ocio activo en el último año si respondió de forma afirmativa a alguna de las preguntas anteriores. Finalmente, esta variable se definió con dos categorías: sí y no.

Se incluyeron variables sociodemográficas como el sexo (hombre y mujer), grupos de edad (18 a 29, 30 a 44, 45 a 59 y más de 60 años), estado civil (casado/conviviente, separado/divorciado/viudo y soltero), nivel educativo (sin educación, primaria, secundaria y superior), identificación indígena “¿pertenece o es descendiente de algún pueblo indígena?” (no y sí); trabajo actual “la semana pasada ¿trabajó al menos una hora, sin considerar los quehaceres del hogar?” (no y sí).

También se consideró a la enfermedad crónica (que incluyó a la presión arterial alta, diabetes, asma o enfermedad alérgica respiratoria, tumor o cáncer, enfermedad renal crónica y SIDA/VIH, diagnosticado por un profesional de la salud): “¿le ha dicho alguna vez un médico u otro profesional de la salud que usted tiene [nombre de la enfermedad o condición de salud]?” (no y sí); enfermedad mental (que incluyó a la depresión y ansiedad diagnosticada por un profesional de la salud): “¿le ha dicho alguna vez un médico u otro profesional de la salud que usted tiene [nombre de la enfermedad o condición de salud]?” (no y sí); deporte: “en el último mes, ¿practicó deporte o realizó actividad física fuera de su horario de trabajo, durante 30 minutos o más?” (no y sí); percepción de discriminación: “¿se ha sentido discriminado? (es decir, se le ha impedido hacer algo, se le ha molestado o se le ha hecho sentir inferior)” (no y sí) y asistencia de un cuidador: “debido a su salud, ¿tiene a alguien que lo ayude en su hogar o fuera de él, incluyendo familiares y amigo, para realizar las siguientes actividades?” (no y sí).

### Fuente de medidas

La ENDISC II emplea un diseño metodológico novedoso basado en la “encuesta modelo de la discapacidad” (EMD) ^15^, diferente a lo habitualmente usado por otras encuestas poblacionales que han empleado los criterios de Washington para medir la discapacidad ^16^. La EMD permite la exploración de la discapacidad como un “*efecto de la acción recíproca entre un sujeto con una condición de salud y diversos factores ambientales y personales*” ^11^. Utiliza escalas continuas para clasificar a la población, basándose en conceptos de capacidad y desempeño que permiten la relación dinámica de la capacidad y de la condición de salud con la influencia de factores ambientales y personales, constituyéndose en un modelo estadístico, diferente a los instrumentos existentes hasta entonces ^11^ ^,^ ^17^.

Para estimar la prevalencia de discapacidad, la ENDISC II usó el método de puntaje de corte empleado en el *Informe Mundial sobre la Discapacidad 2011* ^9^. Dicho método consistió en construir una métrica de funcionamiento que fue de 0 (sin dificultad) a 100 (máxima dificultad), sobre la base de preguntas de los ocho dominios de funcionamiento según la *Clasificación Internacional del Funcionamiento de la Discapacidad y la Salud* (CIF), estableciendo un punto de corte (media de puntuaciones obtenidas de las preguntas sobre dificultades extremas o severas en alguno de los ocho dominios de funcionamiento) que sirvió para determinar el grupo de personas en situación de discapacidad. La media de puntaje obtenido fue de 40 de la escala de 0 a 100 ^14^.

### Análisis estadístico

La base de datos de la ENDISC II se descargó de la página web del SENADIS ^18^ y se analizó con el programa Stata versión 16 (https://www.stata.com). Las variables cualitativas se resumieron en frecuencias y porcentajes. Las diferencias según la participación en las actividades de ocio activo se determinaron con la prueba de chi-cuadrado corregido con el estadístico F, por el diseño muestral. La variación porcentual en las actividades de ocio según discapacidad se calculó con la siguiente fórmula:

$$Variación\%=\frac{\left({\%}_{dis}-{\%}_{no\;dis}\right)}{\left|{\%}_{dis}\right|}\times 100$$

Para estimar la asociación entre la discapacidad y la participación en actividades de ocio activo se usaron modelos crudos y ajustados de regresión de Poisson y se obtuvieron razones de prevalencia (RP) con sus intervalos de 95% de confianza (IC95%) ^19^. Las variables que resultaron asociadas en el modelo crudo (p < 0,05) se incluyeron en el modelo ajustado. La posible existencia de multicolinealidad se evaluó con la determinación del factor de inflación de la varianza (*variance inflation factor* -VIF), asumiendo multicolinealidad cuando el VIF > 5. Se utilizó un diagrama de bosque para visualizar la asociación principal obtenida del modelo ajustado, estratificado por grupos de edad. El diseño muestral complejo se tuvo en cuenta en todos los cálculos y se aceptó un valor de p < 0,05 como significativo.

## Resultados

Un total de 12.265 de personas de 18 a más años participaron en la encuesta. Se excluyeron 29 por datos faltantes en las variables de interés (5 en la variable “nivel educativo”, 9 en la variable “identificación indígena”, 4 en la variable “percepción de discriminación” y 11 en la variable “deporte”), quedando 12.236 personas para el análisis final.

En el análisis descriptivo, el 51,7% era del sexo femenino, el 29,4 % tenía entre 45 y 59 años, el 53,4% estaba casado o era conviviente, el 42,2% tenía un nivel secundario de educación, el 92,2% no se identificaba con ningún grupo indígena, y en el momento de la entrevista el 55,6% tenía un trabajo. Asimismo, el 41,2% tenía una enfermedad crónica y el 11,7% tenía una enfermedad mental, ya sea ansiedad o depresión, el 35,3% había realizado deporte en los últimos seis meses, el 10,5% tenía la asistencia de un cuidador y el 13% había percibido discriminación. Finalmente, el 20% tenía discapacidad, de los cuales el 11,7% tenía discapacidad moderada y el 8,3% tenía discapacidad severa. El 88,2% refirió que había participado actividades de ocio activo en los últimos seis meses (Tabla 1).

**Tabla 1:** Características de las personas de 18 a más años participantes del estudio (N = 12.236).

Características	n	% *	IC95%
Sexo			
Hombre	5.297	48,3	47,0-49,6
Mujer	6.939	51,7	50,4-52,9
Grupos de edad (años)			
18 a 29	2.362	23,4	22,3-24,6
30 a 44	2.957	22,6	21,6-23,7
45 a 59	3.418	29,4	28,2-30,5
Más de 60	3.499	24,6	23,6-25,7
Estado civil			
Casado/Conviviente	6.091	53,4	52,0-54,6
Separado/Divorciado/Viudo	2.398	14,1	13,3-14,9
Soltero	3.747	32,5	31,3-33,8
Nivel educativo			
Sin educación	337	2,5	2,2-2,9
Primaria	3.316	24,8	23,7-25,9
Secundaria	5.160	42,2	40,9-43,5
Superior	3.423	30,5	29,3-31,7
Identificación indígena			
No	11.112	92,2	91,6-92,8
Sí	1.124	7,8	7,2-8,4
Trabajo actual			
No	5.614	44,4	43,1-45,6
Sí	6.622	55,6	54,4-56,9
Enfermedad crónica			
No	6.894	58,8	57,6-60,0
Sí	5.342	41,2	40,0-42,5
Enfermedad mental			
No	10.702	88,3	87,5-89,1
Sí	1534	11,7	10,9-12,5
Deporte			
No	8.115	64,7	63,5-65,9
Sí	4.121	35,3	34,1-36,5
Asistencia de un cuidador			
No	10.871	89,5	88,8-90,3
Sí	1.365	10,5	9,7-11,2
Percepción de discriminación			
No	10.576	87,0	86,2-87,8
Sí	1.660	13,0	12,2-13,8
Discapacidad			
Sin discapacidad	9.622	80,0	78,9-80,9
Discapacidad moderada	1.526	11,7	10,9-12,5
Discapacidad severa	1.088	8,3	7,7-9,0
Participación en actividades de ocio activo			
Sí	10.716	88,2	87,4-89,0
No	1.520	11,8	11,0-12,6

ENDISC II: *II Encuesta Nacional de la Discapacidad*; IC95%: intervalo de 95% de confianza.

* Porcentaje ponderado según el diseño muestral de la ENDISC II.

En el análisis bivariado, se observa que las proporciones de participación en actividades de ocio activo disminuyen de acuerdo con los niveles de discapacidad, pasando de 91,6% en las personas sin discapacidad, 83,2% en las personas con discapacidad moderada y 62,9% en las personas con discapacidad severa, siendo estas diferencias significativas (p < 0,001) (Tabla 2).

**Tabla 2:** Diferencias según la participación en actividades de ocio activo en personas de 18 a más años de Chile (N = 12.236).

Características	Actividades de ocio activo		Valor de p
	Sí	No	
	n (%)	n (%)	
Sexo			0,949
Hombre	4.637 (88,2)	660 (11,8)	
Mujer	6.079 (88,3)	860 (11,7)	
Grupos de edad (años)			< 0,001
18 a 29	2.262 (96,6)	100 (3,4)	
30 a 44	2.736 (92,6)	221 (7,4)	
45 a 59	2.918 (86,1)	500 (13,9)	
Más de 60	2.800 (78,8)	699 (21,2)	
Estado civil			< 0,001
Casado/Conviviente	5.342 (87,6)	749 (12,4)	
Separado/Divorciado/Viudo	1.970 (80,7)	428 (19,3)	
Soltero	3.404 (92,5)	343 (7,5)	
Nivel educativo			< 0,001
Sin educación	219 (62,2)	118 (37,8)	
Primaria	2.582 (76,7)	734 (23,3)	
Secundaria	4.597 (89,8)	563 (10,2)	
Superior	3.318 (97,6)	105 (2,4)	
Identificación indígena			0,415
No	9.716 (88,3)	1.396 (11,7)	
Sí	1.000 (87,2)	124 (12,8)	
Trabajo actual			< 0,001
No	4.750 (85,0)	864 (15,0)	
Sí	5.966 (90,8)	656 (9,2)	
Enfermedad crónica			< 0,001
No	6.296 (92,0)	598 (8,0)	
Sí	4.420 (8,3)	922 (1,7)	
Enfermedad mental			< 0,001
No	9.455 (89,1)	1.247 (10,9)	
Sí	1.261 (81,8)	273 (18,2)	
Deporte			< 0,001
No	6.777 (84,2)	1.338 (15,8)	
Sí	3.939 (95,6)	182 (4,4)	
Asistencia de un cuidador			< 0,001
No	9.749 (90,5)	1.122 (9,5)	
Sí	967 (69,3)	398 (30,7)	
Percepción de discriminación			< 0,001
No	9.331 (88,9)	1.242 (11,1)	
Sí	1.382 (83,7)	278 (16,3)	
Discapacidad			< 0,001
Sin discapacidad	8.741 (91,6)	881 (8,4)	
Discapacidad moderada	1.275 (83,2)	251 (16,8)	
Discapacidad severa	700 (62,9)	388 (37,1)	

Nota: resultados ponderados según diseño muestral de la *II Encuesta Nacional de la Discapacidad* (ENDISC II).

Las diferencias porcentuales significativas según la discapacidad se hallaron en casi todas las actividades recreativas (p < 0,001). Acudir a eventos deportivos y concurrir a bares, discotecas, pubs o fiestas fueron las actividades con mayor diferencia porcentual (64% y 71,2%, respectivamente), es decir, hubo una menor participación de las personas con discapacidad en estos eventos. Ir a festividades o eventos religiosos fue la única actividad en la que no se encontraron diferencias significativas (p = 0,756) (Tabla 3).

**Tabla 3:** Diferencias en la participación en actividades de ocio activo según discapacidad (N = 12.236).

Actividades de ocio activo	Sin discapacidad	Con discapacidad	Variación %	Valor de p *
	n (%) **	n (%) **		
Ir al cine	3.148 (35,6)	346 (14,9)	58,1	< 0,001
Ir al teatro	582 (6,2)	84 (3,1)	50,0	< 0,001
Ir a espectáculos/recitales de música popular, clásica, lírica, ballet, etc.	1.077 (12,0)	148 (5,7)	52,5	< 0,001
Ir a museos o exposiciones	1.183 (13,2)	139 (5,0)	62,1	< 0,001
Ir a eventos deportivos (campeonatos, etc.)	1.661 (18,9)	163 (6,8)	64,0	< 0,001
Ir a festividades o eventos locales (desfiles, rodeos, festivales, etc.)	1.151 (11,3)	152 (6,0)	46,9	< 0,001
Ir a parques/jardines	3.165 (34,5)	459 (18,7)	45,8	< 0,001
Salir de vacaciones	3.011 (33,8)	473 (19,6)	42,0	< 0,001
Ir a actividades al aire libre (acampada, equitación, visitas turísticas, otras actividades al aire libre)	1.944 (21,6)	244 (9,3)	56,9	< 0,001
Ir a eventos de clubes, asociaciones sociales u otros (club deportivo, partido político, centro de madres, etc.)	755 (8,1)	130 (4,9)	39,5	< 0,001
Ir a festividades o actividades religiosas	1.526 (15,4)	429 (15,0)	2,6	0,756
Ir a reuniones sociales o familiares	5.380 (56,5)	1.135 (43,8)	22,5	< 0,001
Ir a restaurantes	3.856 (40,8)	530 (21,5)	47,3	< 0,001
Ir a bares, discotecas, pubs, o “salir de fiesta”	1.934 (22,2)	156 (6,4)	71,2	< 0,001
Ir “a vitrinear” (tiendas, persas, malls, mercados u otros)	5.374 (55,8)	903 (34,6)	38,0	< 0,001
Ir de paseo (dentro o fuera de su localidad)	4.263 (45,0)	691 (25,8)	42,7	< 0,001

Nota: las personas pueden participar en más de una actividad recreativa

* Prueba de chi-cuadrado;

** Porcentaje ponderado según diseño muestral de la *II Encuesta Nacional de la Discapacidad* (ENDISC II).

En el modelo crudo, las personas con discapacidad moderada tuvieron 9% menos probabilidad de participar en actividades de ocio activo (RP = 0,91; IC95%: 0,88-0,94), mientras que, en las personas con discapacidad severa tuvieron 31% menos probabilidad (RP = 0,69; IC95%: 0,64-0,73) en comparación con las personas sin discapacidad. En el modelo final, ajustado por grupos de edad, estado civil, nivel educativo, trabajo actual, enfermedad crónica, enfermedad mental, deporte, asistencia de un cuidador y percepción de discriminación, las personas con discapacidad moderada tuvieron 4% menos probabilidad de participar en actividades de ocio activo (RP = 0,96; IC95%: 0,93-0,99), mientras que, en las personas con discapacidad severa esta probabilidad fue de 22% menor (RP = 0,78; IC95%: 0,72-0,84) en comparación con las personas sin discapacidad (Tablas 4 y 5). No se encontró indicios de multicolinealidad en el modelo final ajustado (VIF ≈ 3).

**Tabla 4:** Asociación entre la discapacidad y la participación en actividades de ocio activo, según análisis multivariado en personas mayores de 18 años de Chile (N = 12.236).

Características	Modelo crudo		Modelo ajustado *	
	RP (IC95%)	Valor de p	RP (IC95%)	Valor de p
Discapacidad				
Sin discapacidad	Referencia		Referencia	
Discapacidad moderada	0,91 (0,88-0,94)	< 0,001	0,96 (0,92-0,99)	0,011
Discapacidad severa	0,69 (0,64-0,73)	< 0,001	0,78 (0,72-0,84)	< 0,001

IC95%: intervalo de 95% de confianza; RP: razón de prevalencia.

Nota: resultados ponderados según diseño muestral de la *II Encuesta Nacional de la Discapacidad* (ENDISC II).

* Ajustado por grupos de edad, estado civil, nivel educativo, trabajo actual, enfermedad crónica, enfermedad mental, deporte, asistencia de un cuidador y percepción de discriminación.

**Tabla 5:** Modelo crudo y ajustado que estima la asociación entre las covariables y la participación en actividades de ocio activo.

Características	Modelo crudo		Modelo ajustado *	
	RP (IC95%)	Valor de p	RP (IC95%)	Valor de p
Discapacidad				
Sin discapacidad	Referencia		Referencia	
Discapacidad moderada	0,91 (0,88-0,94)	< 0,001	0,96 (0,93-0,99)	0,025
Discapacidad severa	0,69 (0,64-0,73)	< 0,001	0,78 (0,72-0,84)	< 0,001
Sexo				
Hombre	Referencia			
Mujer	1,01 (0,98-1,02)	0,950	-	-
Grupos de edad (años)				
18 a 29	Referencia		Referencia	
30 a 44	0,96 (0,94-0,97)	< 0,001	0,99 (0,97-1,01)	0,257
45 a 59	0,89 (0,87-0,91)	< 0,001	0,96 (0,93-0,98)	0,002
Más de 60	0,81 (0,79-0,84)	< 0,001	0,94 (0,91-0,97)	< 0,001
Estado civil				
Casado/Conviviente	Referencia		Referencia	
Separado/Divorciado/Viudo	0,92 (0,89-0,95)	< 0,001	0,98 (0,95-1,01)	0,287
Soltero	1,05 (1,04-1,07)	< 0,001	0,99 (0,97-1,01)	0,216
Nivel educativo				
Sin educación	Referencia		Referencia	
Primaria	1,23 (1,09-1,40)	0,001	1,13 (0,99-1,28)	0,058
Secundaria	1,44 (1,27-1,63)	< 0,001	1,26 (1,11-1,43)	< 0,001
Superior	1,57 (1,39-1,77)	< 0,001	1,34 (1,18-1,52)	< 0,001
Identificación indígena				
No	Referencia			
Sí	0,99 (0,95-1.02)	0,434	-	-
Trabajo actual				
No	Referencia		Referencia	
Sí	1,07 (1,05-1,09)	< 0,001	0,99 (0,98-1,01)	0,444
Enfermedad crónica				
No	Referencia		Referencia	
Sí	0,90 (0,88-0,92)	< 0,001	1,01 (0,99-1,02)	0,500
Enfermedad mental				
No	Referencia		Referencia	
Sí	0,92 (0,89-0,95)	< 0,001	1,01 (0,97-1,04)	0,753
Deporte				
No	Referencia		Referencia	
Sí	1,14 (1,12-1,15)	< 0,001	1,06 (1,05-1,08)	< 0,001
Asistencia de un cuidador				
No	Referencia		Referencia	
Sí	0,77 (0,73-0,81)	< 0,001	0,96 (0,91-1,02)	0,216
Percepción de discriminación				
No	Referencia		Referencia	
Sí	0,94 (0,91-0,97)	< 0,001	0,98 (0,96-1,01)	0,343

IC95%: intervalo de 95% de confianza; RP: razón de prevalencia.

Nota: resultados ponderados según diseño muestral de la *II Encuesta Nacional de la Discapacidad* (ENDISC II).

* Ajustado por discapacidad, grupos de edad, estado civil, nivel educativo, trabajo actual, enfermedad crónica, enfermedad mental, deporte, asistencia de un cuidador y percepción de discriminación.

**Figura 1:**
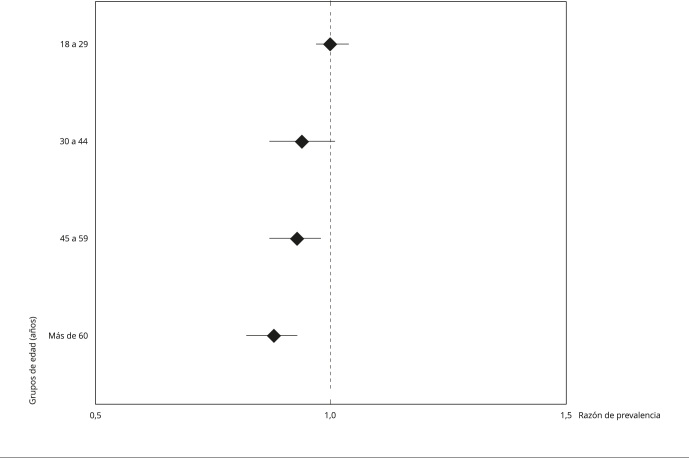
Asociación entre la discapacidad y la participación en actividades de ocio activo (modelo ajustado) estratificado según grupos de edad.

Cuando el modelo final se estratificó por grupos de edad, se observó que las probabilidades de participar en actividades de ocio activo se mantuvieron significativas solo en los grupos de 45 a 59 años y más de 60 años (Figura 1).

## Discusión

Este estudio demuestra que la mayoría de las personas sin discapacidad participaron en actividades de ocio activo en los últimos seis meses, sin embargo, esta cifra fue menor en las personas con discapacidad. Acudir a bares, *pubs* y discotecas o eventos deportivos fueron las actividades con menor participación. Acudir a eventos religiosos fue semejante entre las personas con o sin discapacidad. Las probabilidades de participar en actividades de ocio activo fueron menores en las personas con discapacidad, pero solo si eran mayores de 45 años.

El 88,2% participó en actividades de ocio activo en los últimos seis meses. Esto indica una concurrencia mayoritaria en actividades recreativas de la población chilena. Un análisis de una encuesta de salud de Dinamarca en 55.185 personas mayores de 16 años que tomaron parte en la *Danish Capital Region Health Survey* del 2017 encontró una participación en actividades recreativas basada en la comunidad de solo el 38% ^20^. Esta variabilidad en los reportes supondría que existen factores externos que influyen en la participación en actividades recreativas, y muy posiblemente estén relacionados con el clima, crisis políticas, entre otras; aunque la evidencia es limitada al respecto.

Se encontró que el 74,8% de las personas con discapacidad participaron en actividades de ocio activo en los últimos seis meses. Al respecto, un estudio chileno de diseño transversal realizado en el 2019, que incluyó a 275 personas con discapacidad de 14 programas deportivos, reportó que el 71,4% prefería participar en actividades deportivas-recreativas, es decir, en actividades relacionadas con el disfrute, enseñanza y participación ^21^. Un estudio cualitativo en personas con discapacidad física de 30 a 59 años que pertenecían al Departamento de la Discapacidad de Concepción en Chile encontró una participación significativa en actividades de ocio y tiempo libre en la comunidad, pero solo en actividades concebidas exclusivamente para personas con discapacidad ^22^. Asimismo, un estudio transversal que incluyó a 1.521 personas con discapacidad de 15 a 64 años que se incluyeron en la segunda ola de la *Panel Survey of Employment for the Disabled* (PSED) de Corea del Sur, reportó una participación en actividades sociales del 93% (poca participación en el 32,4%, moderada participación en el 54,1% y alta participación en el 6,5%) ^23^. Una encuesta transversal realizada en 55 sobrevivientes de accidentes cerebrovasculares de Nigeria en el 2014, que incluyó 34 actividades de ocio, encontró una prevalencia de participación en estas actividades del 89,1% ^24^. Los pocos trabajos sobre este tema han mostrado una mayor participación de las personas con discapacidad en actividades de ocio, aunque estos estudios incluyen actividades activas y pasivas. De acuerdo con la revisión de estos trabajos, se puede concluir que el porcentaje de participación en actividades recreativas está de acuerdo con lo descrito en estudios similares.

En general, las personas con discapacidad tuvieron menor participación en casi todas las actividades recreativas, destacando una menor concurrencia a eventos deportivos y en bares, *pubs*, discotecas o fiestas. Al respecto, un estudio ejecutado en 237 adultos con discapacidad del desarrollo (discapacidad intelectual y parálisis cerebral) de España, que utilizó la versión española de Leisure Assessment Inventory que incluye la medición de 53 actividades de ocio, encontró una menor asistencia a eventos deportivos y a conciertos ^25^. De igual forma, un estudio realizado en adultos jóvenes con discapacidad intelectual, mostró menos participación en eventos deportivos, ir al cine u otro entretenimiento similar ^26^, lo que apoya los hallazgos del presente estudio. Al parecer, las situaciones que demandan mayor exposición social, es decir, mayor contacto con personas, limitan la participación de las personas con discapacidad. Se han descrito barreras como la discriminación percibida o el estigma como posibles causas, sobre todo en las personas con discapacidad visual y auditiva ^27^.

No obstante, acudir a festividades o actividades religiosas resultó ser semejante entre las personas con o sin discapacidad. En este caso, la discapacidad no condiciona a una menor participación, como si ocurre en las otras actividades. Sobre esto, algunos autores han reportado hallazgos similares ^28^ ^,^ ^29^, aunque la evidencia es limitada. Una posible explicación estaría referida a la posibilidad de una “cura milagrosa” de la condición que ocasiona la discapacidad; sin embargo, se debe ser muy cauteloso con esta afirmación. Resulta necesario la realización de estudios primarios que consideren a la religión como un factor que podría influir en los aspectos sociales y sanitarios de la discapacidad.

Como hallazgo principal, este estudio encontró que las personas con discapacidad tuvieron menor probabilidad de participar en actividades de ocio activo en comparación con las personas sin discapacidad y que estas probabilidades disminuyen conforme aumenta la severidad de la discapacidad. Algunas investigaciones han descrito resultados semejantes. Un estudio realizado en 11.328 adultos mayores de 65 años de Estados Unidos, que analizó los datos de la *National Health and Aging Trends Study* (NHATS) desde el 2011 al 2015, encontró que las personas con discapacidad tenían menos probabilidades de participar en actividades significativas (actividades placenteras que están asociadas con intereses personales) y esto aumentaba cuando las personas tenían demencia y depresión ^12^.

Esta asociación también puede explicarse de forma reversa, es decir, que las actividades de recreación podrían prevenir o disminuir la discapacidad. Al respecto, un estudio longitudinal efectuado en 12.331 mayores de 80 años que participaron en la *Chinese Longitudinal Healthy Longevity Survey* (CLHLS), una encuesta que evaluó los determinantes del envejecimiento saludable desde 1988 al 2014, percibió que participar en actividades de ocio se asoció con un menor riesgo de discapacidad de las actividades de la vida diaria, independientemente de variables sociodemográficas, estilos de vida y enfermedades crónicas ^29^. Asimismo, un estudio que utilizó cuatro oleadas (1993, 1997, 2003 y 2007) de la *Taiwan Longitudinal Study on Aging* (TLSA), una encuesta de envejecimiento de Taiwán realizada en 5.451 adultos mayores, halló que la participación en actividades de tiempo libre tiene un efecto tres veces mayor en la desaceleración de la progresión de las discapacidades funcionales ^30^. De igual forma, en una cohorte basada en la comunidad de Nara, en Japón, que incluyó a 8.275 residentes de 65 a más años sin discapacidad al inicio del estudio, se encontró una asociación significativa entre las actividades de ocio más frecuentes, medido con 14 actividades de ocio (activo y pasivo) y un menor riesgo de discapacidad incidente, tanto física como cognitiva ^31^.

Posibles explicaciones sobre la menor participación en actividades de ocio activo estarían relacionadas con diversas barreras como la falta de dinero, dificultades en el transporte y la poca información de las ofertas de recreación para personas con discapacidad ^32^. Asimismo, el estado de salud también es una importante barrera hallada en algunos estudios ^13^ ^,^ ^32^. Además, se ha descrito que las personas con discapacidad priorizan la realización de actividades sedentarias, que no promueven la salud ^13^ y que son solitarias y pasivas ^25^; esto sobre todo en los adultos mayores ^33^. Sin embargo, estas limitantes se han reportado en países desarrollados, por lo que es esperable que puedan variar en países de Latinoamérica. Futuros estudios primarios deberían identificar estas barreras, a fin de que puedan ser consideradas en las políticas de salud orientadas a mejorar la calidad de vida de las personas con discapacidad.

Esta asociación solo estuvo presente en los grupos de edad de 45 a 59 años y en los de más de 60. La mayoría de los estudios que han demostrado esta asociación se han realizado en adultos mayores ^12^ ^,^ ^13^, con resultados semejantes al nuestro. Al parecer, en los demás grupos de edad la realización de actividades de ocio activo no parece estar influenciada por la discapacidad. Es decir, la participación en estas actividades sería semejante entre los jóvenes con o sin discapacidad. Este hallazgo es importante porque permitiría enfocar los programas de actividades recreativas hacia las personas mayores con discapacidad.

### Limitaciones y fortalezas

El presente análisis tiene limitaciones propias del diseño. Primero, la medición de la discapacidad con preguntas autorreferidas podría incrementar la prevalencia final de esta condición, debido al sesgo de deseabilidad social o de recuerdo, sin embargo, este sesgo está presente en todos los estudios que recopilan información a través de encuestas. Segundo, podría existir confusión residual, ya que otras variables que podrían estar asociadas con la participación en actividades de ocio activo no estaban disponibles en la ENDISC II, no obstante, se han incluido variables sociodemográficas y relacionadas con las actividades recreativas como confusores importantes. Tercero, debido a la antigüedad de los datos, esta asociación podría cambiar en la actualidad, sobre todo, luego de la pandemia de la COVID-19; sin embargo, no hemos encontrado estudios similares en Latinoamérica, por lo que este sería uno de los primeros en abordar este problema con la presente metodología. Cuarto, la medición de la participación en actividades de ocio activo no fue realizada con un instrumento estándar; es decir, que las preguntas utilizadas pueden variar entre estudios similares, por esta razón, las comparaciones deben realizarse con cautela. Quinto, no se efectuó el análisis según tipo de discapacidad, por lo que se desconoce si alguna de ellas tiene mayor influencia en la participación en actividades recreativas. Sexto, debido a la ausencia de temporalidad, no se puede afirmar causalidad entre las variables principales. Como fortaleza se debe reconocer que la muestra analizada es representativa de toda la población chilena. Se recomienda que futuros estudios tengan un enfoque primario, incluyan variables de confusión reconocidas, utilicen una escala validada para medir las actividades de ocio e incorporen la variable tiempo para evaluar causalidad, a fin de sopesar las limitaciones del presente estudio.

## Conclusiones

La discapacidad se asocia con una menor participación en actividades de ocio activo en la población chilena, lo que confirma nuestra hipótesis. Asimismo, las probabilidades de participar en actividades de ocio activo disminuyen conforme aumenta la severidad de la discapacidad. Esta asociación solo estuvo presente en los mayores de 45 años. La menor participación ocurrió en eventos deportivos y en bares, *pubs*, discotecas o fiestas. Sin embargo, la discapacidad no afectó la participación en actividades religiosas. Es necesario que futuros trabajos primarios determinen las barreras que limitan el acceso a las actividades recreativas en esta población vulnerable. Los resultados del presente estudio podrían orientar a los decisores en políticas de salud a implementar programas de participación social y de actividades de ocio, enfocados en las personas mayores con discapacidad.
